# Hydroxychloroquine Attenuates Atherosclerosis in Apolipoprotein E Knockout Mice: Role of Endothelial Nitric Oxide Synthase and Hypoxia-Inducible Factor 1-Alpha

**DOI:** 10.14740/cr2186

**Published:** 2026-04-15

**Authors:** Eirini Poulakida, Maria Ioannou, Dimitrios Sagris, Erietta Polychronopoulou, Peter K. Makaritsis, Spiros Georgopoulos, Andrew Xanthopoulos, Evangelos Kouvaras, Kassiani Kapatou, Eftichia Asprodini, Ioannis A. Nanas, Athanasios Konstantinidis, George Κ. Koukoulis, George Ν. Dalekos, Konstantinos P. Makaritsis

**Affiliations:** a Department of Medicine & Research Laboratory of Internal Medicine, Faculty of Medicine, University of Thessaly, National Expertise Center of Greece in Autoimmune Liver Diseases, General University Hospital of Larissa, Larissa, Greece; b Department of Pathology, Faculty of Medicine, School of Health Sciences, University of Thessaly, Larissa, Greece; c Biomedical Research Foundation of the Academy of Athens, Athens, Greece; d Department of Cardiology, Faculty of Medicine, School of Health Sciences, University of Thessaly, Larissa, Greece; e Laboratory of Pharmacology, Faculty of Medicine, School of Health Sciences, University of Thessaly, Larissa, Greece; f Department of Obstetrics & Reproduction, Faculty of Veterinary Science, School of Health Sciences, University of Thessaly, Karditsa, Greece; g Hellenic Republic, Region of Thessaly, Division of Veterinary Medicine, Larissa, Greece

**Keywords:** Apolipoprotein E deficient mice, Atherosclerosis, Hydroxychloroquine, Endothelial nitric oxide synthase, Hypoxia-inducible factor-1 alpha, Lipids

## Abstract

**Background:**

We aimed to test the effect of hydroxychloroquine (HCQ) treatment on atherosclerosis and plasma lipids in apolipoprotein E deficient (ApoE^−/−^) mice, defining the aortic expression of endothelial nitric oxide synthase (eNOS) and hypoxia inducible factor-1 alpha (HIF-1α).

**Methods:**

Forty-seven (47) mice were divided into two treatment groups: an HCQ group administered 10 mg/kg/day in drinking water for 16 weeks and a control group with no HCQ. All mice were maintained on a standard chow diet containing 5% fat and had free access to water. At 32 weeks of age, blood was drawn for plasma lipid determination and the proximal aorta was removed to measure the atherosclerotic area and evaluate the expression of eNOS and HIF-1α by immunohistochemistry.

**Results:**

The HCQ group consisted of 16 mice (10 males, six females), while the control group consisted of 31 mice (17 males, 14 females). HCQ significantly reduced the atherosclerotic area (mm^2^ ± SEM) in treated mice compared to controls, both in males (0.0456 ± 0.0140 vs. 0.1920 ± 0.0284, P < 0.001, respectively) and females (0.0278 ± 0.005 vs. 0.1765 ± 0.025, P = 0.003, respectively). eNOS expression was significantly increased, whereas HIF-1α expression was significantly decreased in the aortas of HCQ-treated male and female mice compared to controls. No significant reduction in plasma cholesterol levels was observed in HCQ-treated mice compared with controls.

**Conclusion:**

HCQ reduces aortic atherosclerosis in ApoE^−/−^ mice, potentially through modulation of eNOS and HIF-1α expression, without exerting a beneficial effect on plasma cholesterol levels.

## Introduction

Hydroxychloroquine (HCQ) initially used as an antimalarial drug, has been enlisted during the last decades as a beneficial medication in several autoimmune diseases [1]. It is well known that patients with rheumatoid arthritis and systemic lupus erythematosus develop atherosclerosis more frequently than the general population [2]. It has been also shown by retrospective studies that HCQ might be useful for the reduction of cardiovascular events in these patients [3, 4]. However, data on the effect of antimalarial drugs on atherosclerosis are limited by low consistency and a lack of specifically designed studies, with the overall quality of evidence rated as very low. In a systematic review of eight studies, five reported no significant effect of current or past HCQ treatment on the presence of atherosclerosis [5].

Atherosclerosis stands as the primary contributor to illness and death on a global scale [6]. A clear connection has been established between irregular lipid levels, oxygen deficiency (hypoxia), and impairment of endothelial function in the progression of atherosclerosis. Numerous molecules have been associated with the onset of atherosclerosis [7–9]. Nevertheless, the intricate relationship between hypoxia and the functionality of endothelial cells in inflammatory conditions and atherosclerosis remains complex and lacks a comprehensive understanding [10]. Endothelial nitric oxide synthase (eNOS) plays a crucial role in governing endothelial function by facilitating the generation of nitric oxide (NO), a potent vasodilator [11]. Hypoxia-inducible factor 1-alpha (HIF-1α), a transcription factor that regulates cellular responses to oxygen deprivation, is involved in several pathways relevant to atherosclerosis, including effects on endothelial function and inflammation [8]. Since their first announcement by Zhang et al, it was shown that mice lacking the apolipoprotein E gene develop spontaneous hypercholesterolemia along with atherosclerotic arterial lesions histopathologically similar to those observed in humans [12]. Because of their aforementioned characteristics, the apolipoprotein E deficient (ApoE^−/−^) mice are considered as the classic experimental model for the study of atherosclerosis. In this context, a study by our team in ApoE^−/−^ mice showed that treatment with high dose of HCQ at 100 mg/kg/day led to increased atherosclerosis by overexpression of HIF-1α and eNOS in the aorta of treated mice [13, 14]. In addition, a study of HCQ administration in ApoE^−/−^ mice with chronic kidney disease has shown attenuation of atherosclerosis without any impact of the drug on inflammation markers or the lipid profile of the treated mice [15]. Moreover, antimalarial artesunate ameliorates atherosclerosis by inhibiting inflammasomes activation. Although artesunate and HCQ are antimalarial drugs with distinct mechanisms of action, both have been investigated for potential vascular and atheroprotective effects [16–18].

In the present study, HCQ was administered for 16 weeks to ApoE knockout mice at a dose of 10 mg/kg/day. At 32 weeks of age, the effect of the drug on the atherosclerosis area of the ascending aorta was determined. Since eNOS and HIF-1α are considered to play a significant role in the development of atherosclerosis, the expression of these two molecules at the atherosclerotic plaque was also evaluated by immunohistochemistry [12, 19, 20].

## Materials and Methods

### Drug treatment

HCQ (10 mg/kg/day) was given in the drinking water. The appropriate concentration of HCQ to achieve the target dose was estimated twice a week according to the body weight (BW) increase (from 24 to 34 g/mouse) observed in control and HCQ-treated mice throughout the 16-week treatment period. The HCQ aqueous solution was prepared every 2–5 days and was always kept in dark by wrapping the drinking bottle with aluminum foil. The average volume of water consumed per mouse per day was estimated at approximately 5 mL. HCQ solution was prepared using 200 mg tablets (200 mg/tablet) supplied by Sanofi Co.

### Laboratory animals (mice)

ApoE^−/−^ male and female mice, backcrossed for 10 generations to C57BL/6J, were purchased from Jackson Laboratories, Bar Harbor, Maine, USA. Mice were delivered to our facility at 8 weeks of age and acclimatized for 8 weeks under controlled environmental conditions (12:12 h light–dark cycle, 21 ± 2 °C). Forty-seven (47) mice were randomly divided into two treatment groups and allocated to receive either HCQ at 10 mg/kg/day in the drinking water (n = 16; 10 males, six females) or plain water (control group, no HCQ; n = 31; 17 males, 14 females) for a period of 16 weeks. Because sex-related differences can significantly influence pathophysiological mechanisms and treatment responses, the mice were intentionally stratified into male and female groups to ensure methodological rigor and improve the validity of the findings. Sex-related differences in atherosclerosis development and endothelial function have been well documented in ApoE^−/−^ mice and other experimental models [21]. Moreover, National Institutes of Health guidelines emphasize the importance of including sex as a biological variable in preclinical research [22]. All mice were fed a standard chow diet containing 5% fat and had free access to water. All experiments were conducted according to international guidelines and national (Animal Act, P.D. 160/91) and international laws and policies (EEC Council Directive 86/609, JL 358, 1; December 12, 1987, and UK Animals (Scientific Procedures) Act, 1986 and associated guidelines, EU Directive 2010/63/EU for the care and use of laboratory animals; NIH Guide for Care and Use of Laboratory Animals, NIH publication no. 85-23, 1985) and approved by the Committee for Animal Care of the University of Thessaly, School of Health Science, Faculty of Medicine and the Veterinary Office for Animal Welfare, Drugs and Veterinary Applications Division, Region of Thessaly, Greece (Ethic Committee Name: Veterinary Science Department, Region of Thessaly, Greece, Approval Code: 593752, Approval Date: December 13, 2024).

### Evaluation of atherosclerotic area

At 32 weeks of age and after a 16-week treatment period, an abdominal incision was performed under general anesthesia with intraperitoneal ketamine and blood was collected from the inferior vena cava for plasma lipid measurement. Subsequently, the left ventricle was cannulated, the mouse was perfused with phosphate-buffered saline (PBS) and then with 4% acetate-buffered formalin. Then, the mice were euthanized with an overdose of ketamine. The heart and proximal aorta were removed, weighed, and kept in formalin overnight. The whole procedure for measuring the aortic atherosclerotic area was first described in detail by Paigen et al [23]. The heart was sliced with a scalpel to align the aortic root for subsequent sectioning and the tissue was embedded in paraffin using conventional procedures. Sections were cut at 3 µm using a Leica TP1020 microtome (Leica Biosystems, Illinois, USA) and stained with standard hematoxylin and eosin stain. Sections were obtained from the region starting with the aortic sinus; five sections were reserved for morphometric analysis according to Paigen et al instructions [23]. Sections were viewed using light microscopy and were photographed at an original magnification of × 40 using a Nikon DS-5M-L1 Digital Sight Camera System (Nikon Instruments Inc., New York, USA) and analyzed using the public domain software for image analysis (Image J for microscopy). The extent of atherosclerosis (lesion area) was first delineated using an area selection tool and subsequently quantified with the Analyze/Measure command. Measurements were expressed in pixels. For each mouse, data from five sections were averaged and reported as mean lesion area per section in square millimeters (mm^2^).

### Plasma lipid determination

Blood samples were used for measurement of total cholesterol, low-density lipoprotein (LDL) cholesterol, high-density lipoprotein (HDL) cholesterol, and triglycerides. Blood samples were collected into tubes containing ethylene diamine tetraacetic acid (EDTA) as an anticoagulant and immediately centrifuged to separate plasma. Measurement of plasma lipid levels was performed using an automated chemistry analyzer (AU2700 Chemistry Analyzer, Beckman Coulter, California, USA).

### Immunohistochemistry

#### eNOS immunohistochemistry

Immunohistochemistry was performed using a primary antibody against eNOS (mouse monoclonal antibody, clone 6H2; Novus Biologicals, Littleton, CO, USA) at a dilution of 1:50 for 30 min at room temperature. Detection was carried out using the EnVision polymer–peroxidase system (EnVision/HRP; Dako, Denmark), followed by a 30-min incubation. Bound antibodies were visualized with 0.05% 3,3′-diaminobenzidine (DAB; Dako), and sections were counterstained with hematoxylin and mounted in dibutylphthalate polystyrene xylene (DPX; BDH, UK). Human normal placenta sections served as positive controls, while negative controls were processed by omitting the primary antibody.

#### Evaluation of eNOS immunostaining

Slides were evaluated in a blinded manner. eNOS staining was both membranous and cytoplasmic, primarily localized in macrophages within atherosclerotic plaques. Immunoreactivity was assessed using a semiquantitative scoring system based on staining intensity and the percentage of the stained area within the entire plaque section. The percentage of positive staining was rated as follows: 1 point for < 1–25%; 2 points for 26–50%; and 3 points for > 50%. Staining intensity was scored as follows: 0 points for no staining; 1 point for weak; 2 points for moderate; and 3 points for strong intensity [24]. The scores for staining intensity and area were summed to provide a total score, and specimens were classified into two groups: group 1 (low eNOS expression; total score 0–3) and group 2 (high eNOS expression; total score 4–6).

#### HIF-1α immunohistochemistry

Following antigen retrieval, sections were cooled and rinsed in PBS, then incubated overnight at 4 °C with the primary antibody against HIF-1α (clone H1alpha67; Novus Biologicals; dilution 1:25). Detection was performed using the EnVision polymer detection system (Dako) for 30 min. Bound antibodies were visualized with DAB, and sections were counterstained with hematoxylin and mounted in DPX. A renal cell carcinoma tissue sample from a patient with von Hippel–Lindau syndrome, known to express HIF-1α, was also used as a positive control. Negative controls were processed by omitting the primary antibody and substituting it with nonimmune serum.

#### Evaluation of HIF-1α immunostaining

Slides were evaluated in a blinded manner, and a total quantitative HIF-1α immunoreactivity score was determined for each case using the “Q score” (Quick score) method [24, 25], which is calculated by multiplying the extent and intensity scores. Multifocal or diffuse nuclear immunostaining observed in macrophages throughout the entire plaque was assigned an extent score of 100. Staining present in approximately half of the plaque received a score of 50, while staining in one-fifth of the plaque was scored as 20, with intermediate values assigned proportionally. Staining intensity was assessed relative to the positive control, assigned a score of 3. Very low staining visible only at high magnification was scored as 1, while intermediate intensity as 2. The resulting Q score ranges from 0 to 300.

The same immunohistochemistry protocol used to evaluate eNOS and HIF-1α expression in aortic atherosclerotic lesions of ApoE^−/−^ mice has been previously applied and validated in a prior study conducted by our research group [20].

### Statistical analysis

All values are expressed as mean ± SEM. Differences in BW, plasma cholesterol, LDL cholesterol, HDL cholesterol, triglycerides, lesion area, and eNOS and HIF-1α immunostaining were analyzed using one-way analysis of variance (ANOVA). *Post hoc* comparisons were performed using a two-tailed unpaired Student’s *t*-test. P-values less than 0.05 were considered statistically significant.

## Results

### Effect of HCQ treatment on BW and plasma lipid levels

Table 1 shows BW and plasma lipid profile in ApoE^−/−^ control mice and mice treated with HCQ (10 mg/kg/day) at 32 weeks of age, at the end of the 16-week treatment period.

**Table 1:** Body Weight and Plasma Lipid Profile in ApoE^−/−^ Control Mice and Mice Treated With Hydroxychloroquine 10 mg/kg/day at 32 Weeks of Age, at the End of the 16-Week Treatment Period

	ApoE^−/−^ males		P	ApoE^−/−^ females		P
	Control	HCQ-treated		Control	HCQ-treated	
Body weight (g)	31.60 ± 1.11	33.75 ± 0.49	0.237	30.58 ± 1.47	28.78 ± 0.75	0.546
Total Chol (mg/dL)	449.00 ± 42.29	574.30 ± 45.85	0.061	529.67 ± 20.00	581.66 ± 22.80	0.131
LDL Chol (mg/dL)	364.54 ± 44.70	493.20 ± 41.44	0.049	453.40 ± 15.98	502.50 ± 20.27	0.086
HDL Chol (mg/dL)	61.70 ± 4.69	66.90 ± 4.39	0.429	58.92 ± 3.26	66.16 ± 2.86	0.173
Triglycerides (mg/dL)	113.80 ± 19.92	71.30 ± 8.92	0.067	86.75 ± 8.46	64.66 ± 8.47	0.120

ApoE^−/−^: apolipoprotein E deficient; Chol: cholesterol; HCQ: hydroxychloroquine; HDL Chol: high-density lipoprotein cholesterol; LDL Chol: low-density lipoprotein cholesterol.

There was no difference in BW between control (31.60 ± 1.11 g) and HCQ-treated (33.75 ± 0.49 g) male and female ApoE^−/−^ mice (30.58 ± 1.47 vs. 28.78 ± 0.75 g).

Plasma total cholesterol, HDL cholesterol, and triglyceride levels were not significantly different between control and HCQ-treated mice in both male and female ApoE^−/−^ mice (Table 1). However, HCQ-treated male ApoE^−/−^ mice exhibited significantly higher LDL cholesterol levels compared to controls (493.20 ± 41.44 vs. 364.54 ± 44.70, respectively, P = 0.049) at the end of the 16-week treatment period (Table 1).

### Effect of HCQ treatment on atherosclerosis

**Figure 1:**
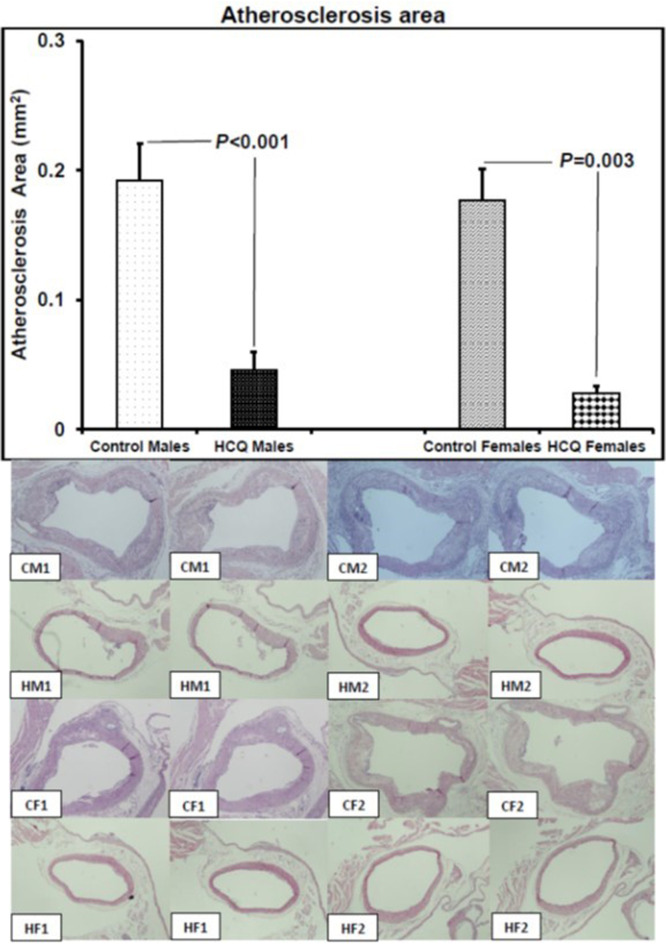
Atherosclerosis area and representative aortic sections (hematoxylin and eosin stain, original magnification × 20) showing atherosclerotic lesions in two male and two female ApoE^−/−^ control and hydroxychloroquine-treated (10 mg/kg/day) mice respectively, at 32 weeks of age, at the end of the 16-week treatment period. CM1: control male #1; CM2: control male #2; HM1: hydroxychloroquine-treated male #1; HM2: hydroxychloroquine-treated male #2; CF1: control female #1; CF2: control female #2; HF1: hydroxychloroquine-treated female #1; HF2: hydroxychloroquine-treated female #2. The left to right images are two representative histological sections, from the junction of the aorta to the heart and beyond into the ascending aorta up to the aortic arch, and used for histopathological evaluation of the entire lesion of each mouse. On histology, the atheromatous plaque of the control male and female mice is thicker than that of the hydroxychloroquine-treated male and female mice. All values are expressed as mean ± SEM. ApoE: apolipoprotein E.

HCQ treatment significantly reduced the aortic atherosclerotic area in both male and female ApoE^−/−^ mice (control vs. HCQ-treated males: 0.1920 ± 0.0284 vs. 0.0456 ± 0.0140 mm^2^, P < 0.001; control vs. HCQ-treated females: 0.1765 ± 0.0250 vs. 0.0278 ± 0.0056 mm^2^, P = 0.003) (Fig. 1). Consistent with previously published studies using ApoE^−/−^ mice as a model of atherosclerosis, lesions composed of foam cells and fibrous plaques were fully developed by 32 weeks of age (Fig. 1).

### Effect of HCQ treatment on eNOS expression

**Figure 2:**
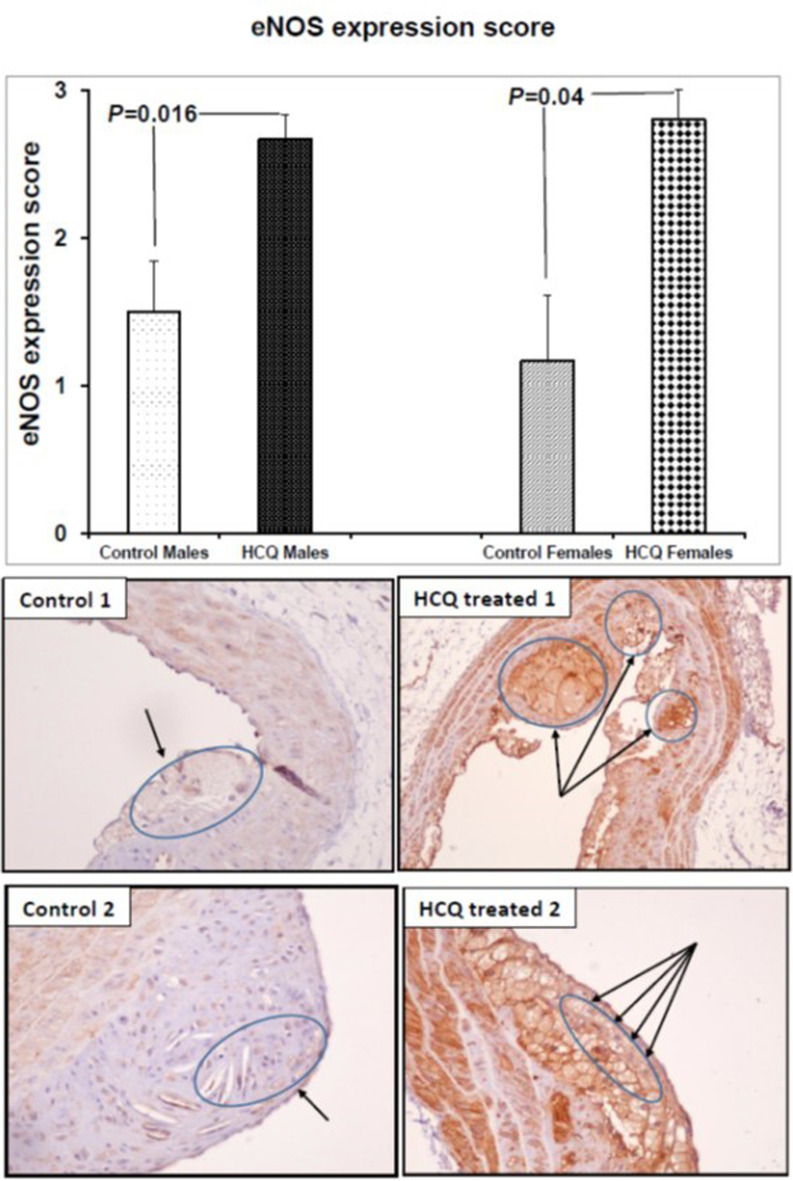
eNOS expression score and representative eNOS immunostained aortic sections (× 20) in two ApoE^−/−^ control and HCQ-treated (10 mg/kg/day) mice at 32 weeks of age, at the end of the 16-week treatment period. Immunohistochemically, the macrophages in the atheromatous plaque of the HCQ-treated mice show strong eNOS immunostaining (long arrows in right panel), whereas the immunoreaction is faint in the control mice plaque (short arrows in left panel). All values are expressed as mean ± SEM. ApoE: apolipoprotein E; eNOS: endothelial nitric oxide synthase; HCQ: hydroxychloroquine.

HCQ treatment increased eNOS expression in the aortic atherosclerotic area of both male and female mice (control vs. HCQ-treated males, 1.50 ± 0.34 vs. 2.67 ± 0.17, respectively, P = 0.016; control vs. HCQ-treated females, 1.17 ± 0.44 vs. 2.80 ± 0.20, respectively, P = 0.04). eNOS immunostaining was strong in the macrophages of the HCQ-treated mice, whereas the immunoreaction was weak in the control mice plaque (Fig. 2).

### Effect of HCQ treatment on HIF-1α expression

**Figure 3:**
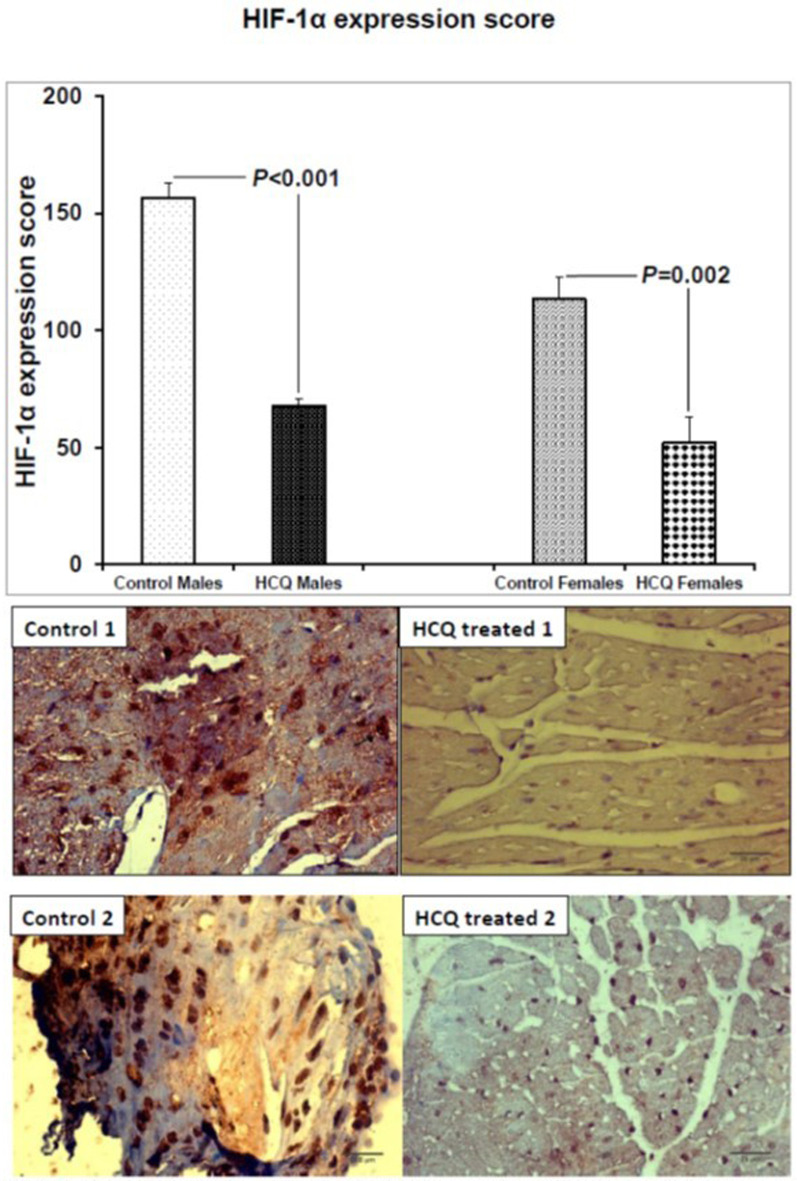
HIF-1α expression score and representative HIF-1α immunostained aortic sections (×40) in two ApoE^−/−^ control and HCQ-treated (10 mg/kg/day) mice at 32 weeks of age, at the end of the 16-week treatment period. Immunohistochemically, few macrophages in the atheromatous plaque of the HCQ-treated mice show weak nuclear HIF-1α immunostaining (blue nuclear staining in right panel), whereas the immunoreaction is strong in almost all nuclei of control mice atheromatous plaque (brown nuclear staining in left panel). All values are expressed as mean ± SEM. ApoE: apolipoprotein E; HCQ: hydroxychloroquine; HIF-1α: hypoxia-induced factor-1 alpha.

HCQ treatment significantly decreased HIF-1α expression in the aortic atherosclerotic area of both male and female mice (control vs. HCQ-treated males, 156.5 ± 6.67 vs. 67.78 ± 2.78, respectively, P < 0.001; control vs. HCQ-treated females, 113.3 ± 9.50 vs. 52.0 ± 11.13, respectively, P = 0.002). Weak HIF-1α immunostaining was demonstrated in the atheromatous plaque of the HCQ-treated mice, whereas the immunoreaction was strong in the macrophage nuclei of control mice (Fig. 3).

## Discussion

The current study demonstrated that a dose of 10 mg/kg/day of HCQ effectively attenuates the progression of atherosclerotic plaques in ApoE^−/−^ mice. HCQ treatment was associated with increased expression of eNOS with concomitant decrease in the expression of HIF-1α in the atherosclerotic plaque. HCQ treatment resulted in atherosclerosis attenuation in this hypercholesterolemic mouse model without affecting the cholesterol, HDL cholesterol, and triglyceride levels of the treated mice. A marginally significant elevation of LDL cholesterol was noticed in male HCQ-treated mice, but this elevation did not diminish the beneficial effect of HCQ treatment on atherosclerosis area (Table 1, Fig. 1). Similar effects with regard to expression of eNOS and HIF-1α have been recently published by our group with the administration of crocin, an active ingredient of saffron, in ApoE^−/−^ mice [20].

Our findings are in agreement with the results reported by Shukla et al [15]. In this study HCQ was administered at the same dosage (10 mg/kg/day) in ApoE knockout mice with chronic kidney disease, fed a high fat diet (21% total fat and 0.15% cholesterol) for 16 weeks. HCQ treatment reduced the area of aortic atherosclerosis by approximately 60%, without also affecting BW, lipid profile, inflammatory markers (high-sensitivity C-reactive protein (hsCRP), interleukin 6 (IL-6), vascular cell adhesion molecule (VCAM)), or renal function. A significant reduction in vascular endothelial dysfunction with improvement in vascular elasticity was also found, but no determination of eNOS or HIF-1α expression was done [15]. In the present study, ApoE^−/−^ mice were fed a chow diet containing 5% fat, rather than the high-fat diet commonly used in the study by Shukla et al [15]. Although a high-fat diet may have increased the atherosclerotic burden in ApoE^−/−^ mice, potentially amplifying the effect of HCQ, our data suggest that HCQ exerts its atheroprotective effects not only in the highly atherogenic environment of ApoE^−/−^ mice fed a high-fat diet but also under conditions of moderate hyperlipidemia. Furthermore, in another study, early treatment with HCQ prevented the development of endothelial dysfunction in a murine model of systemic lupus erythematosus by normalizing endothelium-dependent relaxation and NO availability [26].

Accumulating evidence from experimental models suggests that NO and hypoxia may have a significant role in the progression of the atherosclerotic plaque. It has been well-demonstrated that eNOS expression and subsequently NO exert vasodilating and anti-atherosclerotic effects, including the inhibition of vascular smooth muscle cell proliferation, platelet aggregation, LDL cholesterol oxidation, and vascular inflammation [27, 28]. More specifically, our experiments revealed a significant increase in eNOS expression within the atherosclerotic plaques of mice treated with 10 mg/kg/day of HCQ compared to the control group. These findings align with the observational data related to the plaque size, as reduced plaque size in mice corresponds to an anticipated increase in eNOS expression. This aligns with the typical behavior of eNOS, which tends to exhibit elevated expression in atherosclerotic plaques.

Our group has previously shown that HCQ treatment at higher dosage (100 mg/kg/day) in ApoE^−/−^ mice was associated with unexpectedly accelerated progression of the atherosclerotic plaque, compared to the control group. Interestingly, HCQ treatment at 100 mg/kg/day was associated with a three- to four-fold overexpression of eNOS in the atherosclerotic plaques compared to the control group [13, 14]. However, in the present study, administration of HCQ at a lower dosage of 10 mg/kg/day resulted in a reduction in the size of atherosclerotic plaques, which was accompanied by a small yet significant increase of eNOS. Previous reports have shown that the absence of eNOS accelerates atherosclerotic lesion development in ApoE-deficient mice. It has been shown that overt plaque formation, increased vascular inflammation, and enhanced leukocyte–endothelial (L/E) interactions in ApoE(^−/−^)/eNOS(^−/−^) vessels are associated with a significant reduction in superoxide production. These findings indicate that the absence of eNOS does not automatically result in increased oxidative stress. Although eNOS uncoupling occurs in ApoE(^−/−^) atherosclerosis, this does not negate the overall protective vascular effects of enzyme [29]. In addition, transgenic overexpression of eNOS in ApoE knockout mice resulted in enhanced vascular superoxide production, reduced NO bioavailability, and accelerated atherosclerosis [30]. These findings suggest that a physiological level of eNOS expression may be protective against atherosclerosis, whereas excessive eNOS expression could potentially contribute to lesion progression.

While the exact mechanism by which eNOS overexpression might contribute to atherogenesis remains unknown, the observed expression in eNOS levels in ApoE knockout mice treated at high dose of 100 mg/kg/day of HCQ hints at a potential mechanism involving eNOS overexpression resulting from tetrahydrobiopterin (BH4) depletion and the generation of oxygen free radicals and superoxide. The elevation in eNOS levels in mice treated with a 10 mg/kg/day dosage of HCQ, though still notably higher than in control mice, lends support to this hypothesis.

It has been shown that eNOS activity depends on the availability of BH4, an NOS cofactor. When BH4 levels are inadequate, the reduction of molecular oxygen mediated by the enzyme results in superoxide and oxygen free radicals, rather than NO, production, thereby leading to oxidative stress and endothelial dysfunction. In ApoE knockout mice, endothelium-dependent vascular relaxation is impaired, NO synthesis is reduced, and vascular superoxide production is increased [31, 32]. Tetrahydrobiopterin levels are reduced in the aortas of these mice compared with wild-type (WT) controls, but dietary BH4 supplementation reduces superoxide production and increases NO synthesis [33]. This finding is in agreement with our results in ApoE knockout mice treated with 100 mg/kg/day of HCQ, where eNOS overexpression resulted in accelerated progression of the atherosclerotic plaque in the aortas of treated mice [13, 14]. Co-administration of HCQ at a high dosage of 100 mg/kg/day plus tetrahydrobiopterin supplementation might reverse the harmful effect of HCQ on atherosclerosis and enlighten the exact underlying mechanism [33].

Another noteworthy finding in our study is that the antiatherosclerotic effect of HCQ was linked to a reduction in HIF-1α expression within the aorta of treated mice. Although there might be a pathophysiological association of HIF-1α expression with the progression of the atherosclerotic plaque, the data are scarce. The HIF family of transcription factors is a critical regulator of endothelial function and likely plays a significant role in the atherosclerosis process [34]. HIF-1α is a transcription factor activated under conditions of tissue hypoxia and subsequently triggers the activation of numerous other genes necessary for adapting to a hypoxic environment [35].

Furthermore, in atherosclerotic plaques of ApoE-deficient mice, there has been the identification of hypoxia along with a notable buildup of sterols. This phenomenon was considerably mitigated in laboratory settings by reducing the expression of HIF-1α [36, 37]. Subsequent studies in mice have also revealed the occurrence of hypoxia in atherosclerotic plaques. In a study using atherosclerotic mice of two different genetic backgrounds (LDLR^−/−^ApoB^100/100^) and (IGF-II/LDLR^−/−^ApoB^100/100^), large atherosclerotic plaques were shown to contain hypoxic regions, as evidenced by increased uptake of [^18^F]EF5, a specific marker of hypoxia labeled for positron emission tomography [38]. In another study designed to investigate the role of macrophage HIF-1α in atherosclerosis, LDLR^−/−^ mice were transplanted with bone marrow from mice with HIF-1α deficiency. HIF-1α deficiency in bone marrow-derived cells reduced aortic atherosclerosis in the LDLR^−/−^ recipient mice, suggesting that macrophage HIF-1α expression contributes to the progression of atherosclerotic lesions [39].

The interaction between NO and HIF-1α has gathered significant scientific attention and extensive research [40]. The modulation of the NO/HIF-1α interaction involves intricate molecular pathways, which vary across different tissues and are influenced by factors such as local NO levels, the presence of NO by-products, reactive nitrogen species, and intracellular oxygen levels [10]. The effects of NO on cellular processes differ depending on whether the cells are in a normoxic (adequate oxygen) or hypoxic (oxygen-deprived) state [10]. However, the specific parameters defining normoxia can vary considerably among different tissues, and there is no universally accepted standard for normoxic conditions. Consequently, many studies produce conflicting results, particularly when they use *in vitro* or *in vivo* models. In hypoxic conditions, NO inhibits mitochondrial cytochrome C oxidase, decreases oxygen consumption, and ultimately promotes the degradation of HIF-1α through the proteasome 26S pathway [41, 42]. However, under normoxic circumstances, NO appears to enhance HIF-1α levels and activity [43].

### Limitations

There are specific limitations to our study. Firstly, we fully acknowledge that the use of a murine model represents a major limitation of the study, as findings obtained in mice cannot be directly extrapolated to humans. Animal models, however, provide a controlled experimental environment that allows mechanistic investigation under standardized conditions that would not be feasible in human studies. In addition, the selected mouse model is well established and widely used for studying the biological processes relevant to our research question. However, further studies in humans are required to confirm the translational relevance of our findings. Secondly, we did not precisely determine the daily water and food intake of each mouse, so the dosage of HCQ was based on average measurements made every 3–5 days. However, we took care to prepare consistently the HCQ solution every 2–3 days, and we observed no substantial differences in water consumption between the groups of treated and untreated mice. Furthermore, we did not measure the blood pressure of the mice, which could potentially play a role in the underlying pathophysiology. Lastly, we did not evaluate other markers of inflammation or vascular endothelial dysfunction that might also play a role in atherogenesis.

### Conclusion

In conclusion, we demonstrated that HCQ treatment attenuates the progression of aortic atherosclerotic lesions in both male and female ApoE^−/−^ mice. This atheroprotective effect was accompanied by increased eNOS expression and reduced HIF-1α expression in the aortic plaques of treated mice.

## Data Availability

The authors declare that data supporting the findings of this study are available within the article.

## Abbreviations

ApoB 100: Apolipoprotein B 100
ApoE: apolipoprotein E
BH4: tetrahydrobiopterin
Chol: cholesterol
eNOS: endothelial nitric oxide synthase
HCQ: hydroxychloroquine
HDL Chol: high-density lipoprotein cholesterol
HIF-1α: hypoxia-inducible factor 1-alpha
IGF II: insulin-like growth factor II
hsCRP: high-sensitivity C-reactive protein
IL-6: interleukin 6
LDL Chol: low-density lipoprotein cholesterol
LDLR: low-density lipoprotein receptor
NO: nitric oxide
VCAM: vascular cell adhesion molecule
WT: wild-type
